# Ergotamine Induced Retroperitoneal Fibrosis

**DOI:** 10.31138/mjr.32.2.168

**Published:** 2021-06-30

**Authors:** Susama Patra, Somanath Padhi, Prasanta Padhan, Madhabananda Kar, Prasant Nayak, Subash Chandra Samal

**Affiliations:** 1 Department of Pathology and Laboratory Medicine, All India Institute of Medical Sciences, Bhubaneswar, Odisha, India; 3 Department of Surgical Oncology, All India Institute of Medical Sciences, Bhubaneswar, Odisha, India; 4 Department of Urology, All India Institute of Medical Sciences, Bhubaneswar, Odisha, India; 5 Department of Gastroenterology, All India Institute of Medical Sciences, Bhubaneswar, Odisha, India; 2 Department of Clinical Immunology and Rheumatology, Kalinga Institute of Medical Sciences, KIIT University, Bhubaneshwar, Odisha, India

**Keywords:** RPF, vasculitis, ergotamine

## Abstract

Retroperitoneal fibrosis (RPF) is an uncommon disease characterised by the presence of fibroinflammatory reaction which starts around the infrarenal portion of the abdominal aorta in the retroperitoneum and frequently entrap the ureter causing obstructive uropathy. Approximately, two thirds of the cases are idiopathic, where aetiopathogenesis is not known. Ergotamine-induced RPF, although rare, is considered under secondary group. The fibrogenic process here is thought to be due to serotonergic activity. We report a case of RPF in a young female with obstructive uropathy who had history of long-term ergotamine intake for migraine. Histopathological evaluation revealed different evolving stages of necrotising vasculitis. In addition, the patient has responded to withdrawal of offending drug along with immunosuppressive therapy. We believe, apart from serotonergic activity, ergotamine can lead to RPF through a vasculitic process which has not been reported earlier.

## INTRODUCTION

Retroperitoneal fibrosis (RPF) is a rare fibroinflammatory disorder which is characterised by deposition of fibrous tissue around the abdominal aorta and iliac arteries with a propensity for contiguous involvement of surrounding structures within the retroperitoneal space and frequently causing ureteral obstruction and renal failure.^1^ The annual incidence rate has been reported to be 0.1 to 1.4/1,00,000 subjects in different study populations with more predilection for males than females with a mean age at diagnosis of more than 50 years. This condition has an idiopathic origin in majority of the cases (idiopathic retroperitoneal fibrosis [IRPF]); however, this may arise secondary to malignancy, infection, drugs, chemoradiotherapy, and surgery, or very rarely as a part of Erdheim-Chester disease.^2^ In recent times, the pathophysiology of RPF is better understood, as some of these cases are speculated to be a part of clinically heterogenous immunoglobulin G4 (IgG4) related disease (IgG4-RD).^3^ Therefore, differentiating idiopathic from the secondary RPF is crucial due to completely different therapeutic approach. Although non-contrast enhanced computerised tomogram scan (NCCT) and magnetic resonance imaging (MRI) are useful imaging modalities which help in mapping the extent and severity of fibrosis, these are not useful in differentiating benign versus malignant forms of RPF. Moreover, CT with contrast media is contraindicated in RPF with renal complications.^4^ 18F-fluorodeoxyglucose (FDG-PET) or PET/CT scan helps in diagnosis as well as post therapy monitoring by evaluating the disease activity and its extent.^5^ Histopathological evaluation of a surgical open biopsy specimen is considered as ‘gold standard’ for diagnosis, characterising the true nature of the lesion with atypical presentation such as localised mass-like lesion, confirming IgG4-RD, and monitoring response to immunosuppressive therapy.^1,6^ In this report, we describe a young adult female with histopathologically proven RPF with a history of prolonged ergotamine therapy for migraine and associated vasculitis, which, to the best of our knowledge, has not been described in world literature. Furthermore, the proposed pathophysiologic mechanism of RPF is also briefly highlighted.

## CASE PRESENTATION

**Figure 1.**
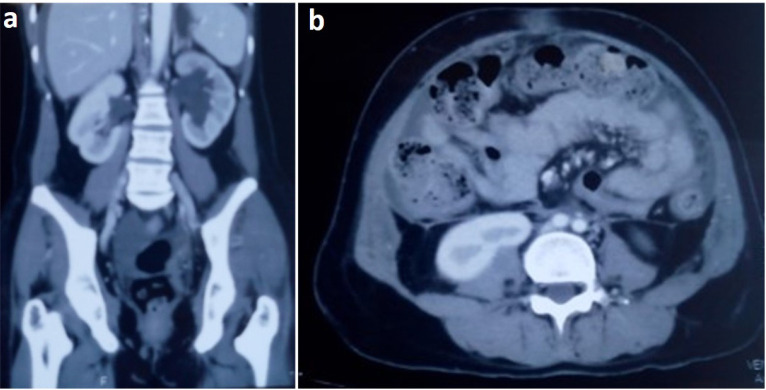
Sagittal (A) and coronal (B) non contrast CT scan of abdomen showing diffuse omental thickening in sub hepatic, infraumbilical, left iliac fossa, and pelvic region with mild thickening of peritoneal layer with mild to moderate ascites; and involvement of bilateral ureters causing mild bilateral hydronephrosis along with mild thickening of ascending colon and prepyloric region.

In December 2017, a 41-year-old female presented with dull aching diffuse pain abdomen of one-year duration. Ultra-sonography (USG) of abdomen and pelvis revealed an ovarian cyst, for which she underwent hysterectomy and bilateral salpingo-oophorectomy at an outside hospital. However, she remained symptomatic after the surgery. On the 7^th^post-operative day, she developed vomiting and was re-evaluated with NCCT of abdomen which showed diffuse omental thickening in sub hepatic, infraumbilical, left iliac fossa, and pelvic region with mild thickening of peritoneal layer with mild to moderate ascites; and involvement of bilateral ureters causing mild bilateral hydronephrosis along with mild thickening of ascending colon and prepyloric region (**Figure 1A,B**). Ascitic fluid cytological analysis showed lymphocyte rich effusion without any evidence of malignant cells based on which she was advised a course of antitubercular therapy (ATT). After 3 months of ATT, there was no improvement and vomiting were persisting. Cartridge-based nucleic acid amplification test (CBNAAT) for tuberculosis was found to be negative; and her serum CA-125 was found to be elevated (166.3 U/ml, ref.; < 30 U/ml) based on which a clinical suspicion of malignancy was raised, and she was referred to our institute for surgical oncology consultation. Upper gastrointestinal endoscopy and colonoscopy did not show any abnormality.

In view of the diagnostic dilemma, she was subjected to exploratory laparotomy of abdomen. Per operatively, omentum was found adhered to pelvis and anterior abdominal wall on multiple bands. Retroperitoneum was hard and fixed with presence of hypervascular fibrosing bands which were seen encroaching small and large bowel mesentery as well as encasing 3^rd^ and 4th part of duodenum causing restricted mobility. In addition, bilateral ureters were also seen to be encased and medially displaced due to the fibrosing bands. Obstructions were relieved by gastro-jejunostomy and jejuno-jejunostomy; and by putting bilateral ureteric double J (DJ) stents. Biopsies taken from omental and peritoneal tissue confirmed the diagnosis of RPF (see below). She was referred to rheumatologist for further work up; and was found to be negative for anti-neutrophil cytoplasmic antibody (ANCA), antinuclear antibody (ANA), anti-ds-DNA, rheumatoid factor, HLA-B27. Her serum IgG4 level was within normal reference limits. Her microbiological testing for viral serology [human immune deficiency virus (HIV), hepatitis B and C] was negative.

On further enquiry, it was found that she was a known case of migraine for which she had been on Vasograin tablet (caffeine + ergotamine + paracetamol + prochlorperazine) for last 20 years. So, a final diagnosis of RPF secondary to prolonged ergotamine use was made. Vasograin was withdrawn and she was started on tablet prednisolone (40mg/day) and mycophenolate mofetil (MMF) (2gm/day). After twelve weeks, MMF was discontinued as the patient developed recurrent vomiting and diarrhoea which was attributed to MMF; prednisolone was tapered slowly to 5 mg alternate days over 6 months and tablet tamoxifen (20mg) was added as an alternative steroid sparing agent with regular imageological follow up. A repeat MRI scan performed 2 ½ years after initiation of therapy showed no significant retroperitoneal soft tissue thickening; normal course of the ureters, and significant reduction in hydronephrosis, more in right side. FDG-PET scan showed mild diffuse thickening around small bowel and peri-pyloric region but without any abnormal metabolic activity or FDG avid lesions. She is presently having a stable disease with alternate day prednisolone and tamoxifen. Her latest follow up abdominal USG (February 2020) showed mild bilateral hydronephrosis with no ureteric DJ stent in place. The sequential laboratory parameters and management details are presented in **Table 1**.

**Table 1.** Sequence of events and laboratory parameters in the index case.

**Month/year**	**12/17**	**01/18**	**3/18**	**5/18**	**8/18**	**9/18**	**11/18**	**1/19**	**3/19**	**8/19**	**2/2020**	**5/2020**
	Diagnosis, stoppage of ergotamine, gastrojejunostomy, jejunostomy, B/L DJ stents in situ	**Steroid + MMF**				**Steroid + tamoxifen, on follow up**						
CRP (mg/dl)		4.5	-	5.0	42.4	1.5	5.0	-	5.36	-	-	4.5
Ur/Cr (mg/dl)		45/0.5	47.4/0.63	-	90/2.0	85/1.8	-	42/1.1	28/1.1	29/1.2	25/1.32	22/1.07
UA (mg/dl)		-	3.47	-	-	-	4.7	5.7	5.2	5.6	-	5.1
Albumin (g/dl)		3.3	-	-	-	-	3.1	3.9	3.6	3.6	-	3.9
Na^+^/K^+^ (mmol/L)		-	140/4.6	-	-	-	142/4.1	137/4.3	140/4.3	142/4.4	-	138/4.5
Ca^2+^/PO_4_^3–^ (mg/dl)		-	10.8/-	-	-	-	9.7/2.9	10.7/3.5	10.1/3.6	9.5/2.5	-	9.6/2.7
Haemoglobin (g/dl)		7.3	-	10	5.75	9.1	-	9.7	9.2	8.6	10.2	-
Leukocyte count^¶^		9700	-	4000	6500	11400	-	6600	6700	9500	WNL	-
Platelet count ^±^		2.8	-	1.9	2.0	1.8	-	1.3	1.4	1.56	WNL	-
ESR		21 mm	-	47mm	102mm	-	11mm	-	32mm	-	-	-
USG abdomen and pelvis						-			B/L DJ stent in situ, mod. hydroureteronephrosis	ND	B/L mild hydroureteronephrosis	-
Magnetic resonance imaging (abdomen, pelvis)	DJ stent										DJ stents taken out, no retroperitoneal soft tissue thickening	-

CRP, C reactive protein; MMF, mycophenolate mofetil; Ur/Cr, serum urea/creatinine; UA, serum uric acid;

¶, per cubic millilitre; ±, lakh per cubic millilitre; ESR, erythrocyte sedimentation rate (/1^st^ hour, Westergren method); –, tests not done, B/L, bilateral; DJ, double J.

## PATHOLOGY

**Figure 2.**
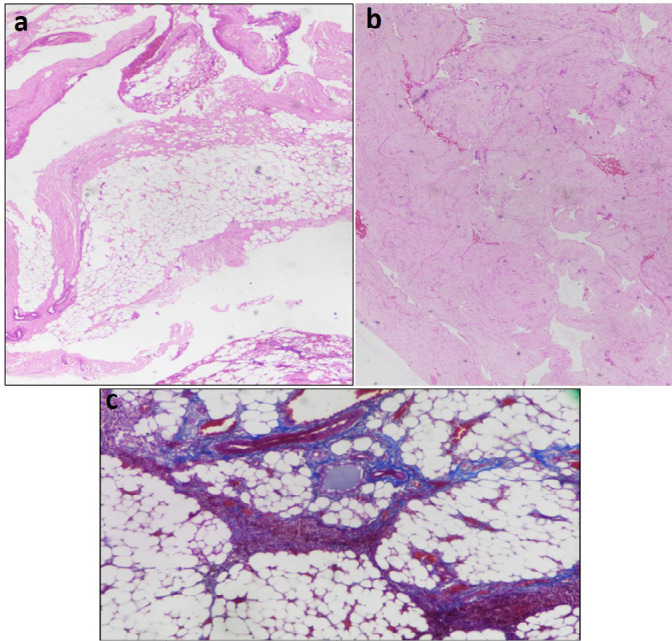
Haematoxylin eosin stained multiple sections from the omental and peritoneal biopsy showing dense collagenous bands of fibrous tissue encircling the lobules of omental fat (A); and at places, with deposition of acellular collagen completely replacing the fat representing old scar (B) (original magnification, x200). Note, the omental fibroinflammatory process at different stages of evolution being highlighted by Masson Trichrome stain (blue) (C, original magnification, x200).

**Figure 3.**
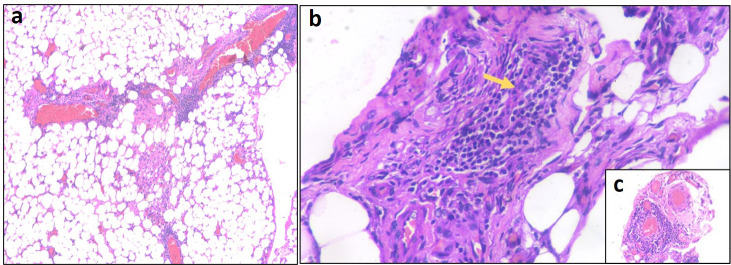
Spectrum of vasculitic changes in omental biopsy: perivascular inflammation (A, x100); evidence of lymphocytic phlebitis characterized by infiltration of venular wall by the lymphoid cells (yellow arrow) (B, x400); circumferential perivenular lymphoplasmacytic infiltration (perivenulitis) (inset*,* C) (Hematoxylin eosin stain). These lymphoid cells were immunopositive for CD 3 and CD 4 (strong); CD 20 (weak); and the plasma cells were negative for IgG4 (not shown in the figure).

**Figure 4.**
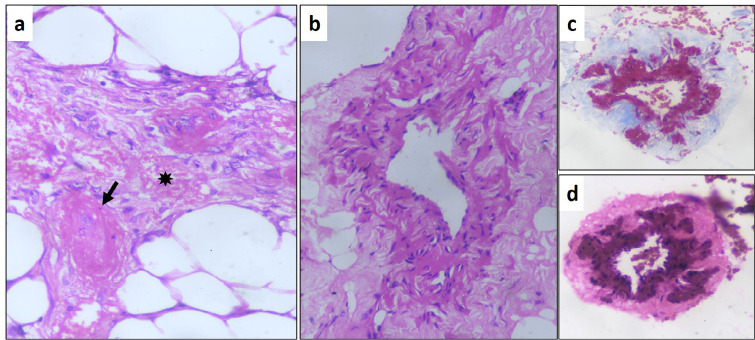
Hematoxylin eosin stained sections highlighting small vessel vasculitis involving arterioles characterized by fibrinoid necrosis of the vessel wall (black arrow) and extravasated red blood cells (black asterisk) (A, x400). Old haemorrhage with hemosiderin laden macrophages were also evident (not shown in the figure). Also note the evidence of healed vasculitis with fibrosis replacing the muscle fibre (B, x400) as highlighted on Masson Trichrome (C, x400) and Verhoeff-Van Gieson (D, x400) stain.

Multiple sections taken from the omentum and peritoneum showed fibrous septae of variable thickness extending from periphery to centre replacing and encircling lobules of adipocytes. The fibrotic process, noted more commonly in peritoneal biopsy, ranged from active fibroblastic proliferation with inflammation to inactive diffuse collagenisation resembling old scar (**Figure 2A,B,C)**. Besides these, patchy areas of vascular congestion and lymphoplasmacytic inflammatory infiltrate were seen mostly around small veins (perivenulitis) with evidence of infiltration of their walls (phlebitis) (**Figure 3A,B,C)**. On immunohistochemistry, the inflammatory infiltrate comprised mainly of CD3^+^, CD4^+^ small mature T lymphocytes admixed with few CD20^+^ B cells. Also, the infiltrating plasma cells were negative for IgG4 monoclonal antibody. The vascular changes ranged from active vasculitis with transmural fibrinoid necrosis to obliterative vasculopathy; whereas healed vasculitis and creeping fibrosis into the vessel wall were more commonly observed in medium sized arterioles (better highlighted by Masson Trichrome and Verhoeff-Van Gieson [VVG] stain) (**Figure 4A,B,C,D**). Foci of old haemorrhage with collection of haemosiderophages were also noted.

## DISCUSSION

The pathophysiologic mechanism of RPF is complex and is poorly understood. Idiopathic retroperitoneal fibrosis (IRPF) is postulated to be a part of chronic periaortitis (CP) that also includes inflammatory abdominal aortic aneurysms (IAAAs) and perianeurysmal retroperitoneal fibrosis (PRF).^2,7–10^ Subsequently, it was observed that all these were different manifestations of the same disease process.^11^ The ‘atherosclerosis theory’ hypothesized that the perivascular inflammation and fibrosis was due to the oxidised low-density lipoprotein (present in atherosclerotic plaque) mediated antigenic presentation to macrophages and subsequent adventitial lymphoplasmacytic inflammation and periadventitial fibrosis.^12,13^ However, occurrence of RPF among children and in individuals without any evidence of atherosclerosis challenged this theory. Moreover, vascular segments which are commonly spared by atherosclerotic process are also involved in RPF pathogenesis. Furthermore, this theory did not support the systemic nature of the disease as has been evidenced by presence of constitutional symptoms; raised serum inflammatory biomarkers and concomitance of other autoimmune conditions such as Hashimoto thyroiditis, Graves disease, or ANCA-associated vasculitis.^6,14,15^

Martorana et al. in their case control study have highlighted the strong association between IRPF and HLA-DRB1^*^03, an allele linked to many autoimmune conditions such as type 1 diabetes mellitus, myasthenia gravis, and autoimmune thyroiditis.^14^ A review of 608 consecutive patients with gastrointestinal (GI) vasculitis from Mayo Clinic, only 5 had evidence of CP at the time of onset of GI symptoms, and none of these were positive for autoimmune markers such as ANA, ANCA, anti-ds-DNA, cryoglobulin, or had any evidence infections such as syphilis, HIV, or Hepatitis B and C.^16^ The presence of necrotising vasculitis and lymphocytic phlebitis; as well as favourable response to immunosuppressive therapy in our case also points towards an immune-mediated vasculitis reaction. Ergotamine related/induced vasculitis was speculated as the possible underlying mechanism in our case though the same was not mentioned as a cause in 2012 Chapel Hill Consensus Conference classification of drug induced vasculitis. ^17^

Drug induced RPF is a rarely reported complication in subjects following the usage of ergot alkaloids (eg, ergotamine, methysergide).^18^ A previous report described a distinctive restrictive type pleuropulmonary pattern of fibrosis following long-term therapy with ergot derivatives (nicergoline, dihydroergocristine, dihydroergotamine), as opposed to typical interstitial pattern observed in other drugs. None of the cases were associated with either clinical or biological evidence of a systemic illness such as rheumatoid arthritis, ankylosing spondylitis, mixed connective tissue disease, lupus erythematosus, or inflammatory bowel disease, and vasculitic mechanism was not described in any of those cases. The outcome was favourable following the withdrawal of ergot derivatives.^19^

To conclude, histopathological demonstration of the entire process of retroperitoneal fibrosis and vasculitis at different stages of evolution with 20-year history of ergotamine ingestion in our case possibly points to a cause and effect relationship. We believe, prolonged ergotamine exposure might have acted like a heptane in this situation to give rise to a small vessel vasculitis culminating in a fibrotic process. The response to immune suppression therapy further substantiates our hypothesis.
